# Violation of the Global Ceasefire in Nagorno-Karabagh: A Viral Amplification of Aggression

**DOI:** 10.1017/S1049023X21000121

**Published:** 2021-01-29

**Authors:** Sharon Chekijian, Alexander Bazarchyan

**Affiliations:** 1. Assistant Professor, Department of Emergency Medicine, Yale School of Medicine, New Haven, Connecticut USA; 2. Director, National Institute of Health of Armenia, Yerevan, Armenia

**Keywords:** Armenia, COVID-19, conflict, Nagorno-Karabagh, pandemic

## Abstract

On March 23, 2020, the United Nations (UN) made an “Appeal for a Global Ceasefire following the Outbreak of Coronavirus.” Despite this appeal, the Nagorno-Karabagh war was instigated on September 27, 2020. This Guest Editorial frames the conflict in the context of the UN appeal and by introducing a figure that plots seven-day average coronavirus disease 2019 (COVID-19) cases overlaid with key inflection points to illustrate the clear impact that conflict has had on pandemic spread in Armenia. The conflict in Nagorno-Karabagh provides a timely, concise, and illustrative example of conflict and its impact on health. Finally, an argument is made that the ability to enforce the UN “Appeal for a Global Ceasefire” is essential to ensure global health and security.

Pandemics notoriously blossom in regions of fragility, conflict, and violence. On March 23, 2020, the United Nations (UN) made an “Appeal for a Global Ceasefire following the Outbreak of Coronavirus.”^1^ The world responded in an unprecedented show of unity. One notable exception stands out. On September 27, 2020, Azeri forces, backed by Turkey, began an unprovoked war in Nagorno-Karabagh or Artsakh.^2^ To wage war during a pandemic is an intentional strategy and may constitute a war crime in and of itself.

Artsakh—located adjacent to the Republic of Armenia and connected by the Lachin corridor—has been home to ethnic Armenians for over 2000 years. Attempts to squash nationalistic sentiments in the USSR resulted in resettlement efforts in Artsakh with groups of Azeri-Turks. The region was administratively under the Soviet Republic of Azerbaijan in name, but remained an autonomous region from 1921 to 1991. Azerbaijan not only willfully neglected the region, but also carried out vicious attacks and ethnic cleansing which intensified as the Soviet Union waned. A defensive war was fought from 1988 to1994. Artsakh carried out a popular vote and was declared an independent state on December 10, 1991 according to the laws governing the dissolution of the Soviet Union.^3^ It has remained an unrecognized Republic since that time. Armenia maintains protective authority over the region. Despite the right to self-determination, Azerbaijan waged war on Artsakh for several reasons, namely in a bid to maintain power domestically.

Since the fall of the USSR, Armenia has flourished, growing from a low-income country to a high middle-income country, according to World Bank classification.^4^ There are many shortcomings of the medical system inherited from the Soviets, which is in the process of systems modernization in both the capital city and the regions. Among other efforts, Armenia has modernized its primary care network, enforced tobacco control, and instituted state of the art cardiac and stroke care.^5-8^ Like many middle-income countries, Armenia had challenges controlling the spread of coronavirus disease 2019 (COVID-19). There was a lack of personal protective equipment, a lack of ventilators, and a lack of testing. Nonetheless, initial efforts to control COVID-19 in Armenia were swift. By September 21, new cases in Armenia totaled 121 per day, down from a high of 771 cases on June 25.^9^ On October 23, after three weeks of war, new cases in Armenia peaked at an astronomical 2,474 in a country of three million people.^9,10^ Armenia became one of the highest growth countries of COVID-19. What is behind this story is a sobering illustration of pandemic growth in the midst of conflict.

As war broke out, massive social mobilization resulted. Young and old gathered to procure supplies for the military. Approximately 100,000 people fled their homes in Artsakh, gathering in bomb shelters and border towns. Thus, COVID-19 flared. As fallen soldiers returned from the front, Yerevan was described as being in a permanent state of funeral proceedings. Each funeral resulted in around 50 to 60 new cases. In turn, each case spread throughout multi-generational households, intensifying the upward trajectory of infections (Figure 1).

**Figure 1.**
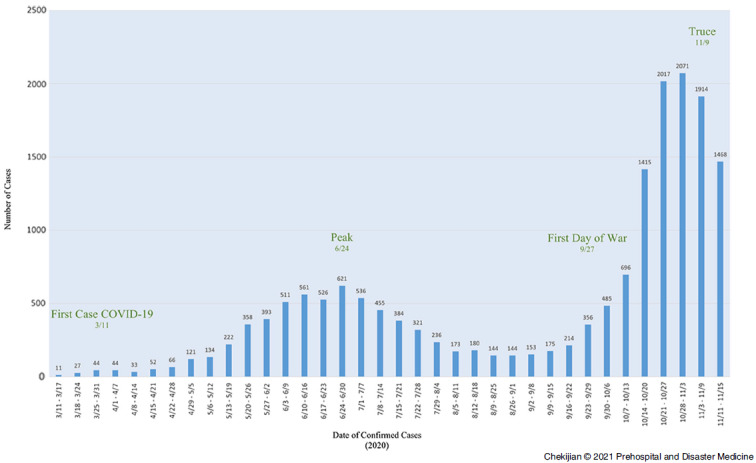
Seven-Day Average of Confirmed COVID-19 Cases in Armenia.

Ambulances, hospitals, and health care workers were targeted in gross violation of the Geneva Conventions. Hospitals that were already stretched, groaned with war injuries and swelling COVID-19 cases. Under normal circumstances, epidemics make provision of medical services extremely hard. Under the non-stop cluster bombings, rocket strikes, and shelling of civilian targets in Artsakh, it was nearly impossible. Sadly, this is exactly what was intended. The syndemic of COVID-19 and war was meant to destroy the self-determination of a people. Despite this, Armenians soldier on as they always have in a quest for survival and self-determination.

Armenians and those concerned for human rights the world over are asking themselves “What good are unenforceable treaties, appeals, and conventions?” These constructs assume “good actors” and a common understanding of civility. The world has changed. Accountability, enforceability, and swift action by the UN Security Council in response to violations of the UN appeal are critical. We need to put teeth into dealing with aggression or we are likely to see it spread more virulently than the virus itself.

## References

[1] The UN has appealed for a global coronavirus cease-fire. *Washington Post.* https://www.washingtonpost.com/politics/2020/04/13/un-has-appealed-global-coronavirus-ceasefire/. Accessed November 6, 2020.

[2] “Then I Heard a Boom:” Heavy Weapons Take Toll on Civilians in Armenia-Azerbaijan Clash. *New York Times*. https://www.nytimes.com/2020/10/05/world/europe/armenia-azerbaijan-nagorno-karabakh.html. Accessed November 6, 2020.

[3] De Waal T. Black Garden: Armenia and Azerbaijan through Peace and War. New York USA: NYU Press; 2013.

[4] World Bank Country and Lending Groups. 2020. https://datahelpdesk.worldbank.org/knowledgebase/articles/906519-world-bank-country-and-lending-groups. Accessed November 6, 2020.

[5] Danielyan KE , Oganesyn HM , Nahapetyan KM , et al. Stroke burden in adults in Armenia. Int J Stroke. 2012;7(3):248–249. 22405280 10.1111/j.1747-4949.2012.00776.x

[6] Harutyunyan A , Abrahamyan A , Hayrumyan V , Petrosyan V. Perceived barriers of tobacco dependence treatment: a mixed-methods study among primary healthcare physicians in Armenia. Prim Health Care Res Dev. 2019;20:e17. 30421696 10.1017/S1463423618000828 PMC6476393

[7] Movsisyan NK , Connolly GN. Measuring Armenia’s progress on the tobacco control scale: an evaluation of tobacco control in an economy in transition, 2005-2009. BMJ Open. 2014;4(2):e004410. 10.1136/bmjopen-2013-004410 PMC3939662 24578541

[8] Petrosyan V , Melkom Melkomian D , Zoidze A , Shroff ZC. National scale-up of results-based financing in primary health care: the case of Armenia. Health Syst Reform. 2017;3(2):117–128. 31514673 10.1080/23288604.2017.1291394

[9] Armenia COVID cases. 2020. https://www.google.com/search?client=safari&rls=en&q=armenia+covid+cases&ie=UTF-8&oe=UTF-8. Accessed October 30, 2020.

[10] COVID Tracker. *New York Times*. https://www.nytimes.com/interactive/2020/world/coronavirus-maps.html#countries. Accessed December 5, 2020.

